# Growth Differentiation Factor-15 in Immunity and Aging

**DOI:** 10.3389/fragi.2022.837575

**Published:** 2022-02-09

**Authors:** Brandt D. Pence

**Affiliations:** College of Health Sciences, University of Memphis, Memphis, TN, United States

**Keywords:** inflammaging, immunosenescence, aging, immunity, GDF-15

## Abstract

Aging increases susceptibility to and severity of a variety of chronic and infectious diseases. Underlying this is dysfunction of the immune system, including chronic increases in low-grade inflammation (inflammaging) and age-related immunosuppression (immunosenescence). Growth differentiation factor-15 (GDF-15) is a stress-, infection-, and inflammation-induced cytokine which is increased in aging and suppresses immune responses. This mini review briefly covers existing knowledge on the immunoregulatory and anti-inflammatory roles of GDF-15, as well as its potential importance in aging and immune function.

## Introduction

Aging is the single largest risk factor for nearly all chronic diseases, including cardiovascular disease, cancer, and neurodegenerative diseases (Niccoli and Partridge, 2012). Underlying nearly all chronic diseases is an increase in inflammation, and numerous observations have associated aging with a chronic low-grade inflammatory state. This has given rise to the term inflammaging, in which age-associated inflammation is suggested to be a shared underlying cause for the progressive decline in physiological function and increased pathology with age (Franceschi and Campisi, 2014). It is now well-appreciated that dysregulated inflammation is closely coupled with the aging phenotype, as inflammatory processes are central to essentially all of the ‘hallmarks of aging’ defined in a landmark paper (Lopez-Otin et al., 2013) published in *Cell* in 2013. Systemic chronic inflammation underlies age-related disease processes across tissue types (Furman et al., 2019) and is predictive of multimorbidity and frailty (Sayed et al., 2021), suggesting that inflammaging is central to biological aging.

In addition to the increase in inflammation seen in the inflammaging state, the aging process also brings about progressive immunosenescence, a generalized decline in immune system function leading to increased complications from infectious diseases and other immunological stimuli (Fulop et al., 2018). Immunosenescence leads to defects in the innate immune system, including impaired phagocytosis and chemotaxis, increased myeloid cell proportion, and altered basal and stimulated cytokine production in granulocytes, monocytes, macrophages, and dendritic cells (Linton and Thoman, 2014; Fulop et al., 2018). Additionally, adaptive immune responses are generally impaired with aging, as lymphocyte subpopulations shift to more regulatory and memory phenotypes, and lymphocyte proliferation and function is decreased (Goronzy and Weyand, 2013; Fulop et al., 2018). Because inflammatory responses are a major aspect of immune regulation, inflammaging and immunosenescence are invariably linked.

However, some aspects of aging and immunity are paradoxical (Montgomery and Shaw, 2015), as anti-inflammatory and immunoregulatory cell subtypes are proportionally increased during the aging process (Fulop et al., 2018), which would be expected to promote a more anti-inflammatory state. Additionally, immune cells are known to take on more pro- or anti-inflammatory roles during aging depending on stimulus, as exemplified by research on monocytes showing age-related increased basal expression of the pro-inflammatory cytokine tumor necrosis factor (TNF)-α (Hearps et al., 2012), while cytokine responses are impaired with aging during inflammatory activation in monocytes (Renshaw et al., 2002; Pence and Yarbro, 2019).

## Senescence

A principal contributor to the aging process is cellular senescence (Lopez-Otin et al., 2013; Yarbro et al., 2020). One of the ‘hallmarks of aging’ (Lopez-Otin et al., 2013), cellular senescence is characterized by proliferative arrest in aging cells, preventing cell division in an irreversible manner (Campisi, 2012). However, despite the similarity in their names, cellular senescence and immunosenescence are different processes with widely-varying outcomes. Immunosenescence refers to a generalized deterioration in immune cell function during aging, predisposing older individuals to worsened outcomes to infectious and chronic diseases (Fulop et al., 2018). While there is some evidence for cellular senescence in the immune system, primarily in lymphocytes (Zhou et al., 2021), mechanisms underlying immunosenescence are not universally driven by hallmarks of cellular senescence such as cell cycle arrest, telomere shortening, etc. Nevertheless, links between cellular senescence and immunosenescence are a promising area of research, as there has been a dramatic increase in interest in the regulation of host processes by senescent cells, driven primarily by the discovery of the senescence-associated secretory phenotype (SASP).

During aging, senescent cells produce a host of secreted factors now known as the SASP, which are involved in the regulation of myriad host functions including immune system-relevant processes such as inflammation, tissue repair, and cellular proliferation (Campisi, 2012). Many SASP factors are cytokines and chemokines which are intimately involved in the regulation of inflammation (Campisi et al., 2011), thus cellular senescence is a primary driver of inflammaging both at local tissue and systemic levels (Freund et al., 2010) and contributes to the pro-inflammatory environment further induced by other age-associated factors such as increases in damage-associated molecular patterns (Kapetanovic et al., 2015). As a major determinant of the host endocrine environment, SASP factors are also prime candidates for potential mechanisms linking cellular senescence and inflammaging to immunosenescence, as many circulating and tissue immune cells are routinely exposed to secreted SASP factors.

## GDF-15

Growth Differentiation Factor-15 (GDF-15) is a distant member of the transforming growth factor (TGF)-β superfamily of cytokines (Unsicker et al., 2013), in that it shares structural characteristics with TGF-β superfamily members but was found to have relatively weak homology with existing superfamily members at the time of its discovery (Bootcov et al., 1997). GDF-15 has detectable expression in nearly all tissues including the brain, intestines, lungs, cardiovascular system, etc. (Yokoyama-Kobayashi et al., 1997; Böttner et al., 1999; Tan et al., 2002).

GDFs have a long history in aging research (Jamaiyar et al., 2017), although this has been controversial due to debates about the direction and effects of age-associated changes to key GDFs including myostatin (GDF-8) and GDF-11 (Loffredo et al., 2013; Katsimpardi et al., 2014; Sinha et al., 2014; Egerman et al., 2015; Smith et al., 2015; Poggioli et al., 2016). However ***,*** the known effects of GDF-15 are distinct from those of GDF-8, GDF-11, and other proteins of this subfamily relevant to aging (Unsicker et al., 2013), and GDF-15 is sufficiently divergent from other TGF-β superfamily members that it was initially suggested to be the first member of a new subfamily of TGF-β-related proteins (Bootcov et al., 1997).

GDF-15 was independently discovered by multiple laboratories in the late 1990s (Bootcov et al., 1997; Hromas et al., 1997; Lawton et al., 1997; Baek et al., 2001), with each laboratory describing a distinct function of the protein. The most influential of these initial publications (by citation count) was from Bootcov *et al.* in 1997 (Bootcov et al., 1997), who named GDF-15 as macrophage inhibitory cytokine-1 (MIC-1) and demonstrated 1) that GDF-15 was released by macrophages due to inflammatory stimuli such as tumor necrosis factor (TNF)-α and interleukin (IL)-1β, and 2) that GDF-15 signaling in macrophages inhibits lipopolysaccharide-stimulated TNF-α production. This seminal manuscript provided evidence that GDF-15 is an important immunoregulatory protein which links an inflammatory state with immunosuppression. Follow-up work from several laboratories has implicated GDF-15 in suppressing function of a variety of immune cells, including neutrophils (Kempf et al., 2011; Artz et al., 2016; Zhang et al., 2016), macrophages (Preusch et al., 2013; Lee et al., 2017; Jung et al., 2018), dendritic cells (Segerer et al., 2012; Zhou et al., 2013; Zhang et al., 2018), natural killer (NK) cells (Roth et al., 2010; Kleinertz et al., 2019), and T lymphocytes (Roth et al., 2010).

In addition to its molecular effects, GDF-15 is well-established as a biomarker for a number of chronic diseases, many of which are increased with age. Increased GDF-15 levels have been associated with cardiovascular disease (Wollert et al., 2017), mitochondrial diseases (Yatsuga et al., 2015), diabetes (Adela and Banerjee, 2015), and cognitive decline (Fuchs et al., 2013) among others. GDF-15 is also an active area of investigation within cancer research, as it has dually-opposing effects including both anti-tumorigenic and pro-metastatic activities depending on cell type studied (Unsicker et al., 2013). Increased GDF-15 has also been suggested as a biomarker for severity of rheumatoid arthritis (Esalatmanesh et al., 2020).

The independent identification by multiple laboratories of GFRAL as the neuronal receptor for GDF-15 (Emmerson et al., 2017; Mullican et al., 2017; Yang et al., 2017) which mediates its known anti-obesity effects has also accelerated research into GDF-15 as a weight-loss promoter. However, the long-term consequences of increasing GDF-15 are questionable, given its association with immunosuppression and various chronic diseases. It is also worth noting that GDF-15 signaling mechanisms are likely to be distinct in different cell types, as canonical TGF-β receptor signaling has been shown to mediate the effects of GDF-15 in various immune cells (Artz et al., 2016; Jung et al., 2018; Zhang et al., 2018; Kleinertz et al., 2019). Importantly, immune cells to do not express GFRAL, necessitating that the observed effects of GDF-15 on leukocytes occur through an alternate receptor.

Further underscoring GFRAL-independent aspects of GDF-15 signaling is the recent observation that GDF-15 activates AMPK in skeletal muscle independent of GFRAL (Aguilar-Recarte et al., 2021). The geroprotector drug metformin also increases circulating GDF-15 levels and promotes weight loss, and GDF-15 knockout abrogates the weight loss effect of metformin in mice (Day et al., 2019). While the GDF-15 mediated weight reduction effects of metformin may be mediated through GFRAL, metformin is also well known as an anti-inflammatory and immunomodulatory drug (Bułdak et al., 2014; Kim et al., 2014; Qing et al., 2019; Soberanes et al., 2019; Cory et al., 2021; Xian et al., 2021), therefore the effect of metformin on inflammation and immune function may be at least partially mediated through promoting GDF-15 expression.

## GDF-15 and Immunity

In addition to suppressing inflammatory responses (Roth et al., 2010; Kempf et al., 2011; Segerer et al., 2012; Preusch et al., 2013; Zhou et al., 2013; Artz et al., 2016; Zhang et al., 2016; Lee et al., 2017; Jung et al., 2018; Zhang et al., 2018), GDF-15 is a potent suppressor of chemotaxis in neutrophils (Kempf et al., 2011; Artz et al., 2016; Zhang et al., 2016), restricts macrophage accumulation in atherosclerotic plaques (Preusch et al., 2013), and promotes autophagy in macrophages (Heduschke et al., 2021). In macrophages, GDF-15 increases reliance on oxidative phosphorylation for energy production and promotes an M2-like phenotype (Jung et al., 2018), which suggests a potential mechanism by which GDF-15 mediates anti-inflammatory responses. GDF-15 also reduces LPS-induced sepsis responses in mice (Abulizi et al., 2017) and suppresses NLRP3 inflammasome activation and inflammatory responses in adipose tissue (Kim et al., 2013; Wang et al., 2014). These findings suggest that GDF-15 is a potent suppressor of inflammatory responses by innate immune cells.

Some evidence suggests that GDF-15 can suppress DC function. In perhaps the most comprehensive study to date, Zhou *et al.* (Zhou et al., 2013) demonstrated impaired expression of maturation markers, reduced inflammatory cytokine production, and impaired T cell activation by GDF-15-treated DCs. These findings are consistent with a study on decidual dendritic cells, which demonstrated impaired maturation and T cell stimulatory capacity in GDF-15-treated DCs (Segerer et al., 2012). GDF-15 has also been shown to promote tolerogenic DC responses, including increasing expression of inhibitory molecules and promoting T cell exhaustion and regulatory T cell production by DCs (Zhang et al., 2018). Aging is known to impair DC function, including by reducing antigen presentation (Wong and Goldstein, 2013). Age-related increases in GDF-15 therefore represents a potential mechanism for the observation of DC impairments during aging.

GDF-15 is also an active area of study in cancer immunity (Wischhusen et al., 2020; Lodi et al., 2021), and GDF-15 has been shown to allow gliomas to evade immune responses by suppressing natural killer cell-mediated immunity and T cell migration into the tumors (Roth et al., 2010). GDF-15 also suppresses macrophage anti-tumor responses during early cancer development (Ratnam et al., 2017). GDF-15 is highly overexpressed in colorectal, ovarian, lung, and many other cancer types (Wischhusen et al., 2020), and is therefore considered a promising biomarker for cancer prognosis in addition to its potential role in cancer cell evasion of anti-tumor immune responses.

GDF-15 is additionally relevant to infectious disease responses. GDF-15 impairs NK cell function during systemic infection (Kleinertz et al., 2019) and regulates polarization of adipose tissue macrophages (Lee et al., 2017). One recent paper demonstrated that GDF-15 overproduction increases severity of human rhinovirus infections (Wu et al., 2017), and Kleinertz *et al.* reported that GDF-15 levels were increased in injury patients who progressed to sepsis compared to those who did not (Kleinertz et al., 2019). Likewise, GDF-15 expression is increased in cells infected with avian influenza viruses, and this in turn limits their production of cytokines (Zhao et al., 2021). COVID-19 patients have elevated GDF-15 levels (Notz et al., 2020; Rochette et al., 2021), suggesting a potential link between GDF-15 and disease severity in the ongoing pandemic.

In septic patients, GDF-15 levels are predictive of disease severity and mortality (Buendgens et al., 2017), giving further evidence that GDF-15 may be of import in systemic immune responses. Finally, GDF-15 has been shown to be released due to infection with a variety of bacterial and viral pathogens, and that it plays a tissue protective role during infection *via* regulation of lipid metabolism (Luan et al., 2019). As such, there is abundant evidence that GDF-15 plays a multifaceted role in immune responses by down-regulating immune function, not unlike impairments to the immune system noted in age-related immunosenescence.

## GDF-15 and Aging

Within the aging field, GDF-15 has very recently become a protein of tremendous interest. Although GDF-15 was identified in 2010 as being associated with all-cause mortality in Swedish males (Wiklund et al., 2010), the protein was largely forgotten in the aging field until a 2018 publication in *Aging Cell* by Tanaka *et al.* (Tanaka et al., 2018) profiled the plasma proteome across the lifespan. They demonstrated that GDF-15 was the protein most strongly associated with age and that it increased in a linear fashion as age increased. Follow-up studies by the same authors indicated that GDF-15 was the protein most strongly associated with multimorbidity, including after adjustment for age and sex (Tanaka et al., 2020).

At least several additional papers support the finding that GDF-15 levels increase with age (Ho et al., 2012; Doerstling et al., 2018), and elevated GDF-15 levels are associated with the development of anemia in older adults (Yamaguchi et al., 2021). Underscoring this, Tavenier *et al.* recently described a strong association between GDF-15 levels and accelerated aging phenotypes in older adults, wherein individuals with frailty had on average an approximately 60% increase in GDF-15 compared to age-matched healthy individuals (Tavenier et al., 2021). This finding supported a previous report linking GDF-15 levels in the plasma to frailty (Conte et al., 2020). Although in need of further support, these findings suggest GDF-15 as potentially prognostic of biological aging.

A recent study by Basisty *et al.* (Basisty et al., 2020) profiled the SASP across multiple cell types and *in vitro* senescence inducers with the goal of developing a “SASP Atlas” to support research in this field. GDF-15 was identified as part of the “core SASP” which was upregulated across cell types and treatments, confirming its importance as potential signaling molecule in cellular senescence. These recent findings underscore the potential importance of GDF-15 to aging, although the actual molecular contributions of GDF-15 to aging are currently unknown.

Given its relatively recent emergence as a biomarker of aging, it is unsurprising that little is known about the contribution of GDF-15 to immunosenescence. My laboratory has recently described correlations between elevated GDF-15 levels and monocyte dysfunction (Pence et al., 2021) in a secondary analysis using data from our previous reports on age-related metabolic and inflammatory deficits in monocytes (Pence and Yarbro, 2018; Pence and Yarbro, 2019). Interestingly, Moon *et al.* (Moon et al., 2020) recently reported that GDF-15 is increased in aging in response to cell-free mitochondrial DNA, and that this limits tissue inflammatory burden. However, GDF-15 also suppressed T cell activation *via* promoting regulatory T cell activity, thereby limiting immune activation. Circulating GDF-15 levels were additionally recently shown to be correlated to accumulation of senescent T cells during aging (Chen et al., 2020). GDF-15 may therefore be a regulatory SASP factor which limits inflammaging at the expense of suppressing immune function.

## Discussion and Conclusion

Aging is associated with a substantial dysregulation of the immune system. GDF-15 is a stress-induced cytokine which is secreted under pro-inflammatory conditions and serves to limit inflammatory activation in many immune cell types. This suggests a potential inflammation-immunosuppression axis driven by GDF-15, whereby the protein is secreted during an inflammatory or immune activation event, and serves as a signal to assist in self-limiting or resolving the initial pro-inflammatory response.

By extension, chronic inflammation as induced by aging or senescence may therefore lead to chronic elevation of GDF-15 levels, leading to sustained suppression of the immune system. Recent evidence indicates that GDF-15 is highly associated with aging and increases across the lifespan, therefore GDF-15 is a strong potential link between inflammaging and immunosenescence. While there is some limited evidence suggesting that GDF-15 regulates immune function during aging, a great deal more work is necessary to convincingly demonstrate this. Nevertheless, GDF-15 is clearly an extremely important immunoregulatory cytokine which also has strong associations with biological aging. As such, further research in this area is likely to uncover additional links between GDF-15 and age-related immune dysfunction.
